# Assessing the Effects of Surface-Stabilized Zero-Valent Iron Nanoparticles on Diverse Bacteria Species Using Complementary Statistical Models

**DOI:** 10.3390/jfb16030113

**Published:** 2025-03-20

**Authors:** Brittany J. Carnathan, Dinny Stevens, Swarna Shikha, Carson Slater, Nathen Byford, Rodney X. Sturdivant, Kuzy Zarzosa, W. Evan Braswell, Christie M. Sayes

**Affiliations:** 1 Department of Biology, Baylor University, Waco, TX 76798, USA; brittany_carnathan1@baylor.edu; 2 Department of Environmental Science, Baylor University, Waco, TX 76798, USA; dinny_stevens1@baylor.edu (D.S.);; 3 Department of Statistical Science, Baylor University, Waco, TX 76798, USArodney_sturdivant@baylor.edu (R.X.S.); 4 Insect Management and Molecular Diagnostics Laboratory, United States Department of Agriculture, Animal and Plant Health Inspection Service, Plant Protection and Quarantine, Science and Technology, Edinburg, TX 78541, USA; evan.braswell@usda.gov

**Keywords:** FeNP, nZVI, polyvinylpyrrolidone, ascorbic acid, cetyltrimethylammonium bromide, antibacterial, statistical modeling

## Abstract

Nanoparticles are proposed as alternatives to traditional antimicrobial agents. By manipulating a nanoparticle’s core and surface coating, antimicrobial effects against various microbial populations can be customized, known as the “designer effect”. However, the antimicrobial properties of nanoparticle core–coating combinations are understudied; little research exists on their effects on diverse bacteria. The antimicrobial effects of surface-stabilized zero-valent iron nanoparticles (FeNPs) are particularly interesting due to their stability in water and ferromagnetic properties. This study explores the impact of FeNPs coated with three surface coatings on six diverse bacterial species. The FeNPs were synthesized and capped with L-ascorbic acid (AA), cetyltrimethylammonium bromide (CTAB), or polyvinylpyrrolidone (PVP) using a bottom-up approach. Zone of inhibition (ZOI) values, assessed through the disc diffusion assay, indicated that AA-FeNPs and CTAB-FeNPs displayed the most potent antibacterial activity. Bacteria inhibition results ranked from most sensitive to least sensitive are the following: *Bacillus nealsonii* > *Escherichia coli* > *Staphylococcus aureus* > *Delftia acidovorans* > *Chryseobacterium* sp. > *Sphingobacterium multivorum*. Comparisons using ordinal regression and generalized linear mixed models revealed significant differences in bacterial responses to the different coatings and nanoparticle concentrations. The statistical model results are in agreement, thus increasing confidence in these conclusions. This study supports the feasibility of the “designer nanoparticle” concept and offers a framework for future research.

## 1. Introduction

Surface-stabilized nanoparticles made of metal cores are postulated to offer numerous advantages over conventional antimicrobial agents. These benefits include fewer adverse effects on mammalian cells, effective antimicrobial action at low concentrations, and stability under a broad range of physiologically and environmentally relevant conditions [1,2,3,4,5]. Large relative surface area makes nanoparticles ideal for targeted drug delivery as therapeutics, functioning either as the carrier or the active agent [6]. Nanoparticles have a variety of tailorable properties contributing to antimicrobial propensities, such as core composition, size, surface coating, and surface charge [7,8,9,10]. Some nanoparticles induce multiple antibacterial modes of action, including destroying the bacterial cellular structure, producing reactive oxygen species (ROS), inhibiting biofilm formation, and disrupting enzymatic processes [11,12,13,14,15]. These modes of toxicity show promise for slowing the evolution of antimicrobial resistance, which causes about 700,000 deaths per year and billions of U.S. dollars in annual economic losses [12,16,17,18].

To combat resistance using nanotechnology, systematically testing designer nanoparticles against diverse bacteria species is critical. “Designer nanoparticle” refers to the ability to design next-generation drugs and drug delivery devices that selectively interfere with cells of interest [19,20,21]. The designer nanoparticle concept for antibacterial applications is based on the phenomenon that bacteria-specific taxonomic characteristics are responsive to engineered nanoparticle characteristics. However, no systematic studies have investigated this idea; few studies have used multiple taxonomically diverse bacteria species in a single study, and none have analyzed the results relative to bacteria features versus nanoparticle properties. Before the scientific community can develop designer nanoparticles for antibacterial applications, systematic evaluations of gradual variations in nanoparticle properties against diverse bacterial species must be performed [22,23,24].

Despite their antibacterial potential, designer nanoparticles’ practical application is still limited. Studies investigating bacterial inhibition across diverse microbial species using surface-stabilized nanoparticles must be significantly expanded. The crucial role of surface coatings in maintaining the stability of zero-valent metal nanoparticles in aqueous suspensions cannot be overstated. Furthermore, exploring the vast array of available coating materials is paramount to realizing the full potential of nanoparticle-based antibacterial agents.

The antimicrobial propensities of zinc, copper, and silver nanoparticles have been studied extensively [25,26,27,28,29,30,31,32,33,34,35]. However, only a handful of reports have been published on the antibacterial activity of zero-valent iron nanoparticles (FeNPs, also known as nZVI) [36,37,38,39,40,41,42,43,44,45]. Furthermore, even less is known about the role of specific nanoparticle surface coatings. While it has been shown that iron can induce various cytotoxic effects, including disruption of the bacterial cell wall, interruption in electron transfer, and radical species formation through Fenton’s reaction, less is known about the differential antibacterial effects of FeNPs stabilized with different surface coatings [38,40,42,46]. It is unknown if an iron nanoparticle’s stabilizing agent affects the treatment’s antimicrobial potency.

The bacteria used in this study are phylogenetically diverse and occupy various environments (Table 1). Niches range from human pathogens to agriculturally relevant plant symbionts, including taxa ubiquitous across environments. Understanding the effect of surface-coated nanoparticles on these diverse bacterial strains will aid the development of iron nanoparticles as antibacterial agents and provide insights into the environmental impact of nanoparticle exposure on commensal bacteria communities.

This paper describes the experimental approach and resultant data probing the effects of surface-stabilized zero-valent iron nanoparticles on diverse bacteria species using complementary statistical models. This study investigates the antibacterial effects of FeNPs capped with three different surface coatings (i.e., L-ascorbic acid (AA), cetyltrimethylammonium bromide (CTAB), or polyvinylpyrrolidone (PVP)). Increasing FeNP exposure doses (1, 2, and 4 μM) were tested on six bacterial taxa (i.e., *Bacillus nealsonii*, *Chryseobacterium* sp., *Delftia acidovorans*, *Escherichia coli*, *Sphingobacterium multivorum*, and *Staphylococcus aureus*) using the Kirby–Bauer disc diffusion assay. Disc diffusion assays assess antibacterial effects by measuring the zone of inhibition (ZOI) around FeNP-impregnated discs. Disc diffusion assays assess antibacterial effects by measuring the zone of inhibition (ZOI) around FeNP-impregnated discs. Often, this assay generates data that does not conform to normality, which is a central assumption of many statistical tests such as ANOVA. Instead, the data generated are often zero-inflated, necessitating more complex modeling for analysis.

Here, we applied ordinal regression and generalized linear mixed models (GLMM) to assess the impact of nanoparticle coating, bacterial species, and nanoparticle dose on antibacterial efficacy. Comparing species, dosage, and coatings can help direct nano-enabled antibacterial research and development efforts.

## 2. Materials and Methods

### 2.1. Reagents

Polyvinylpyrrolidone (PVP, CAS #9003-39-8, MW 40,000) and iron (III) nitrate nonahydrate (Fe(NO_3_)_3_·9H_2_O, CAS #7782-61-8) were purchased from Alfa Aesar (Haverhill, MA, USA). Cetyltrimethylammonium bromide (CTAB, CAS #57-09-0) was purchased from Sigma-Aldrich (St. Louis, MO, USA). L-ascorbic acid (C_6_H_8_O_6_, CAS #50-81-7), sodium borohydride (NaBH_4_, CAS #16940-66-2), and hydrazine hydrate (N_2_H_4_·H_2_O, CAS #10217-52-4) were purchased from Acros Organics (Thermo Fisher Scientific, Waltham, MA, USA). Nutrient broth and 0.5 McFarland standard were purchased from Remel (Thermo Fisher Scientific). Nutrient agar was purchased from Oxoid (Thermo Fisher Scientific).

### 2.2. Nanoparticle Synthesis

Zero-valent iron nanoparticles (FeNPs) were synthesized using a chemical reduction method. They were surface-stabilized with an acid (L-ascorbic acid (AA)), a surfactant (cetyltrimethylammonium bromide (CTAB)), or a polymer (polyvinylpyrrolidone (PVP)). Synthesis techniques for each capped FeNP utilized water as the solvent to promote green chemistry. Ascorbic acid-coated iron nanoparticles (AA-FeNPs) were synthesized by adding 25 mL of 1 M AA dropwise to 25 mL of 10 mM Fe(NO_3_)_3_·9H_2_O heated to 80 °C and stirred vigorously for 17 h. A color change to red-brown was observed at reaction completion [63].

Cetyltrimethylammonium bromide-coated iron nanoparticles (CTAB-FeNPs) were synthesized in ultrapure water with 25 mL of freshly made 0.01 M CTAB and mixed with 25 mL of 1 mM Fe(NO_3_)_3_·9H_2_O. Next, 50 mL of a 50% *v*/*v* mixture of 0.01 M CTAB and 0.08 M hydrazine hydrate (NH_2_NH_2_ (H_2_O)) solution was added. Pale yellow coloring was observed after 3 h of vigorous magnetic stirring [64].

Polyvinylpyrrolidone-coated iron nanoparticles (PVP-FeNPs) were synthesized by adding 40 mL of 0.033 M Fe(NO_3_)_3_·9H_2_O dropwise to 40 mL of 3.5% PVP under vigorous magnetic stirring. The solution was reduced by adding 20 mL of freshly prepared 0.03 M NaBH_4_ dropwise under vigorous stirring. The reaction occurred immediately, and black coloration was observed after bubble formation ceased [65].

### 2.3. Nanoparticle Characterization

Spectroscopic techniques characterized the size, shape, concentration, agglomeration state, and refractive index. Absorption profiles were obtained using a BioTek Epoch 2 spectrophotometer (Winooski, VT, USA). Dynamic light scattering (DLS) was performed to obtain hydrodynamic diameter, dispersity, and zeta potential measurements (ZEN 3690 Nanoseries Zetasizer; Malvern, Worcestershire, UK). Zeta potential indicated the surface charge of the coated nanoparticle when suspended in water. Values are the charge of the nanoparticle across the interface of the electrical double layer, which forms between the nanoparticle and the solvent.

### 2.4. Bacterial Cultures

Six bacterial lineages were used for the study. *E. coli* 25922 and *S. aureus* 6538 were purchased from ATCC (St. Cloud, MN, USA). *B. nealsonii*, *Chryseobacterium* sp., *D. acidovorans*, and *S. multivorum* were a generous gift from Dr. Don Vacek, USDA, PPQ, APHIS, S&T. The characteristics of each bacterium are described in Table 1.

### 2.5. Antibacterial Disc Assay

The agar disc diffusion assay, or Kirby–Bauer test, was performed to determine the antibacterial efficacy of each coated FeNP on the six bacterial species. Each bacteria colony was transferred into a tube with 10 mL nutrient broth, incubated overnight at 37 °C, and shaken at 100 rpm [8]. Nutrient agar and broth were prepared according to manufacturer instructions. Bacteria were plated at 1 × 10^8^ CFU/mL using the 0.5 McFarland standard [66]. Three dosing concentrations were prepared (1, 2, and 4 mM) for each FeNP type. The nanoparticle-loaded discs were prepared by impregnating 8 mm plain discs with each nanoparticle suspension on cultured Petri plates and tested in triplicate using three discs per plate. The diameter of the disc and zone of inhibition (ZOI) were measured for each replicate. The difference between the ZOI and disc diameter was used to determine antibacterial efficacy. Environmental contamination was controlled by placing FeNP-impregnated discs on agar plates without bacteria and incubating them alongside cultured Petri plates.

### 2.6. Statistical Methods Overview

**Figure A1:**
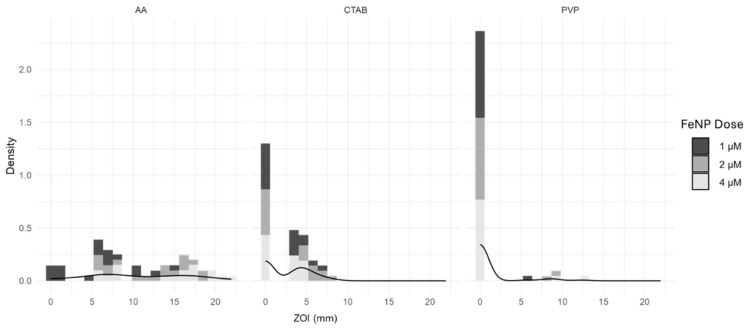
Histograms for coatings and dose. For two-way ANOVA to be viable, the distribution shape would need to resemble a traditional bell curve, and the sample size would need to be far greater than the size *n* = 162.

Traditional tests assessing significance between treatments include assumptions that may not be met when comparing nanoparticle treatments that elicit different antibacterial responses. In this study, the response variable (i.e., bacterial growth inhibition) has a right-skew distribution for all three FeNPs (Figure A1). This deviation from normality egregiously violated assumptions for traditional statistical methods (e.g., ANOVA) and revealed the need for alternative model development.

First, an initial logistic regression was performed, comparing the probabilities of all three FeNP types to induce an antibacterial response [67]. The results of this test confirmed that PVP-FeNP did not cause significant antibacterial activity; thus, it was not included in the subsequent models as it would have decreased the statistical accuracy.

Second, two distinct statistical models were developed: a parametric generalized linear mixed model (GLMM) and a semi-parametric ordinal regression model. Each analysis revealed significant differences between treatment groups (i.e., bacteria species [species], nanoparticle dose [dose], and particle surface coating [coating]).

### 2.7. Generalized Linear Mixed Model Development

The GLMM model was chosen for its ability to describe zero-inflated distributions [68]. It is considered an advanced and more parsimonious two-way ANOVA that extends the traditional generalized linear regression model by adding mixed effects to the fixed effects to capture variation within groups. By using a link function, the model can specify conditional distributions that are not normal. The choice of link function determines the interpretation of the coefficients. These data’s best distribution and link function choices were the hurdle gamma distribution and the log link function. The hurdle-gamma distribution can be considered a two-component distribution with a probability assigned to observing a zero and a scaled likelihood of observing a non-zero value [69]. This can be shown as:

(1) $$f\left(y\right)=\left\{\begin{array}{c} 1-\pi \;\;\;\;\;\;\;\;\;\;\;\;\;\;\;\;\;\;\;\;\;\;\;\mathrm{if}\;y=0,\\ \pi \times gamma\left(y|\lambda ,\nu \right)\;\mathrm{if}\;y>0. \end{array}\right.$$

The variables included in the model are shown in Table 2.

### 2.8. Ordinal Regression Model Development

**Figure A2:**
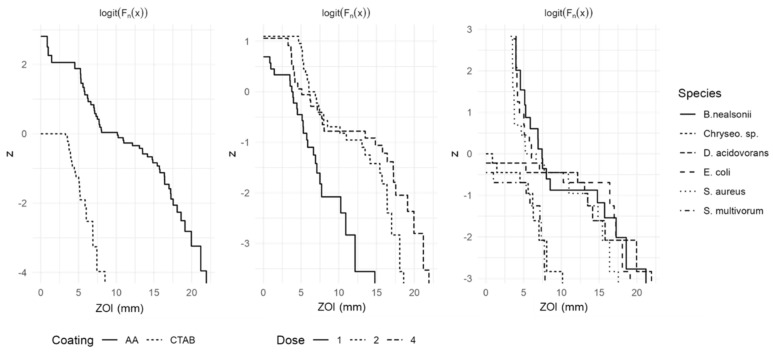
Graphics illustrating the proportional odds for each factor and its levels. The y-axis is the empirical cumulative density function with a logistic link function. Lines that are relatively parallel in the center of the distribution are considered to have proportional odds.

Ordinal regression was chosen as a complementary approach to the GLMM analysis, which lacks distributional assumptions [70] and offers confirmation of the GLMM output. For the ordinal assumptions in this dataset, see Appendix A (Figure A2). If the GLMM and ordinal models agree, we can confidently use the GLMM for inference. Although the two models have different interpretations, they validate each other if they insinuate the same conclusions.

The ordinal regression model estimates the log-odds inhibition ratio of higher versus lower values for a specific treatment relative to the baseline treatment. The baseline treatment information for this model remained the same, i.e., Coating = AA, Species = *S. multivorum*, and Dose = 1 µM. If Y represents the bacterial zone of inhibition with j treatments, then $\mathrm{logit}(P(Y\leq j))=\log\left(\frac{P(Y\leq j)}{P(Y>j)}\right)$ represents the log odds of Y being less than or equal to a specific treatment j. The model can be written as follows:

(2) $$\mathrm{logit}\left(P(Y_{j}\leq j)\right)=\alpha_{j0}+\beta_{1}x_{1}+\cdots +\beta_{8}x_{8}+\beta_{9}x_{1}x_{7}+\beta_{10}x_{1}x_{8}$$

where $\mathrm{logit}\left(p_{i}\right)=\log\left(\frac{p_{i}}{1-p_{i}}\right)$, the log odds ratio of a given treatment induced a greater inhibition than the base treatment. The variables correspond to the same ones in Table 2, except for the no error (ε) term since the response is an odds ratio describing the probability of an increase or decrease response to each treatment.

## 3. Results and Discussion

### 3.1. Iron Nanoparticle Characterization

**Figure 1:**
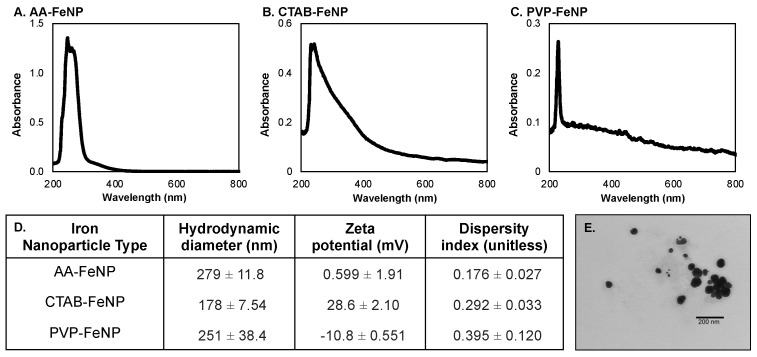
Summary of iron nanoparticle (FeNP) characterization. Panels (**A**–**C**) show the UV–vis spectra for (**A**) AA-FeNP, (**B**) CTAB-FeNP, and (**C**) PVP-FeNP. Table (**D**) summarizes the hydrodynamic diameter, zeta potential, and dispersity index measurements. Panel (**E**) shows a representative TEM image of AA-FeNP (experimental details are included in the Appendix A).

Iron nanoparticles were characterized using UV/Vis spectroscopy and dynamic light scattering (DLS) (Figure 1). AA-FeNP and CTAB-FeNP exhibited absorption maxima at 245 nm, while the absorption maxima for PVP-FeNPs was 230 nm. These results are consistent with previously reported zero-valent FeNP characterization properties [71,72,73]. The hydrodynamic diameter and zeta potential of the FeNP were determined using DLS. The hydrodynamic diameter of AA-FeNPs was 279 ± 12 nm; that of CTAB-FeNPs was 178 ± 8 nm, and that of PVP-FeNPs was 251 ± 38 nm. The surface charge of the nanoparticles was assessed from zeta potential. AA-FeNP was considered neutral with a zeta potential of 0.6 ± 1.9 mV, CTAB was cationic with a zeta potential of +28.6 ± 2.1 mV, and PVP-FeNP was slightly anionic with a charge of −10.8 ± 0.6 mV. Dispersity index values of < 0.400 indicate that particle size was relatively uniform across all FeNP types [74]. The increasing shoulder of the CTAB-FeNP and PVP-FeNP spectra peaks in comparison with AA-FeNP could be attributed to an increase in particle dispersity, which is known to shift absorption of nanoparticle solutions [75].

Previous studies have revealed each capping agent to be effective in forming and stabilizing metallic nanoparticles. Ascorbic acid is a reducing agent during particle core formation and a capping agent to maintain particle stability [76,77]. CTAB is a positively charged surfactant with a hydrophilic head group and hydrocarbon tail, which coats particles and hinders aggregation [78,79]. PVP causes steric hindrance between particles, preventing agglomeration, and can function as a reducing agent during particle formation [80,81].

### 3.2. Coating and Dose Effect on Bacterial Inhibition

**Figure 2:**
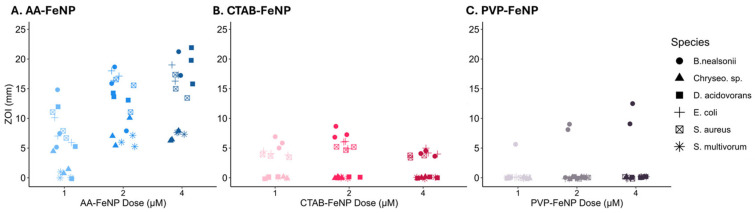
Antimicrobial response to each treatment comparing nanoparticle surface coatings and nanoparticle dose. Zone of inhibition (ZOI) values are grouped by coating and dose across species for (**A**) AA-FeNP, (**B**) CTAB-FeNP, and (**C**) PVP-FeNP exposures. Graphs show the influence of dose and coating on antimicrobial effect across species. Results show that ascorbic acid nanoparticles were most effective at inhibiting bacteria growth.

The antibacterial effects of each FeNP particle type were assessed by the zone of inhibition (ZOI) diameter (Figure 2). AA-FeNPs exhibited the strongest antibacterial effect of the three coatings tested, with a higher collective ZOI diameter than CTAB and PVP coatings. CTAB-FeNPs showed a larger collective ZOI than PVP-FeNPs, which showed little antibacterial activity. Logistical regression output shows a significant increase in the probability of AA-FeNP and CTAB-FeNP inducing an antibacterial effect compared to PVP-FeNP (Table 3).

The diverse antimicrobial propensities of each coating may be due to electrostatic or non-bonding (i.e., steric) interactions. The negative charge of PVP may repel particles from the negatively charged bacteria membrane, leading to the low antimicrobial propensity seen for PVP-FeNP in this study [8]. While CTAB confers a positive charge, the decreased antimicrobial propensity in comparison with the neutrally charged AA could be caused by van der Waals forces or steric interactions; AA has multiple polar groups, which could interact with the polar groups on the bacteria surface to a greater degree than CTAB or PVP [82]. Further, the steric hindrance of the pyrrolidone ring in the PVP polymer and the long hydrocarbon tails of CTAB may hinder interaction with the bacteria membrane.

### 3.3. Dose Effects on the ZOI Varied Based on FeNP Surface Coating

**Figure 3:**
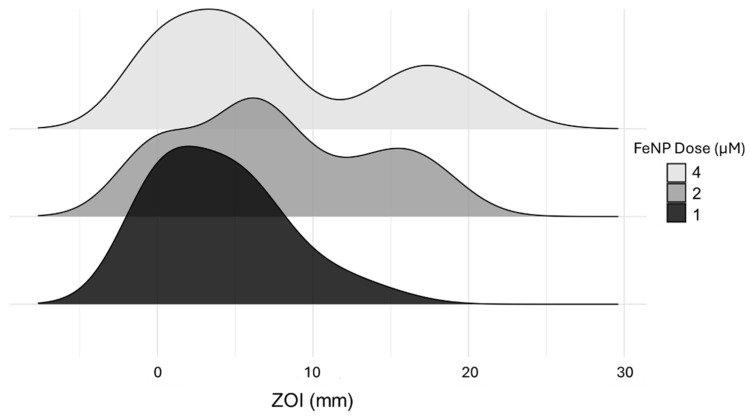
AA-FeNP and CTAB-FeNP zone of inhibition (ZOI) probability distribution grouped by dose. The top, middle, and bottom panels represent joint density results for 4, 2, and 1 µM doses. As stated previously, PVP significantly differs from the other two FeNP types. Results show similar ZOI distributions between 2 and 4 µM treatments.

Graphical analysis of inhibition measurements grouped by dose and coating across species shows that the antibacterial effects of AA-FeNPs were dose-dependent. In contrast, the inhibition response to CTAB-FeNPs was not dose-dependent. The effects of the dose on bacterial growth inhibition for PVP-FeNP were unclear in the collective analysis of this coating. Density plots of compiled AA-FeNP and CTAB-FeNP data show a marked change in distribution between 1 and 2 µM doses, but the distribution remained similar between 2 and 4 µM doses (Figure 3). The absence of a ZOI increase with increasing dose may be due to nanoparticle agglomeration, which could affect particle interaction with the bacteria [83]. The low effective dose of AA-FeNPs and CTAB-FeNPs further demonstrates their usefulness as antibacterial agents.

The antibacterial effects of each surface coating alone have been tested previously, although studies investigating the antibacterial impact of coated FeNPs are limited. This is the first study testing the antibacterial effects of AA-FeNPs, PVP-FeNPs, or CTAB-FeNPs. Previous studies investigating the antibacterial effects of bare (uncapped) zero-valent FeNPs revealed oxidative stress and membrane alteration as dominant modes of action [38,40,42,84]. AA is an antioxidant with antibacterial properties [85,86]. Here, we demonstrate that AA-FeNP is a highly effective antibacterial agent, with ZOI values surpassing 20 mm. The surfactant CTAB is also known to have antibacterial properties apart from a metallic core. However, greater antibacterial efficacy was associated with a metal core, such as silver and copper nanoparticles [8]. PVP is not known to have antibacterial effects apart from its association with nanoparticle metal cores. The variation in efficacy resulting from chemical coatings shows promise for multiple nano-bio applications, including developing effective, safe, and economical solutions to antibacterial resistance and other areas of biomedicine. More studies investigating the relationship between ROS production and the antioxidant capacity of the AA-FeNPs should be designed and reported.

### 3.4. Intra-Species Analysis Revealed Differences in Response to FeNP Coating

**Figure 4:**
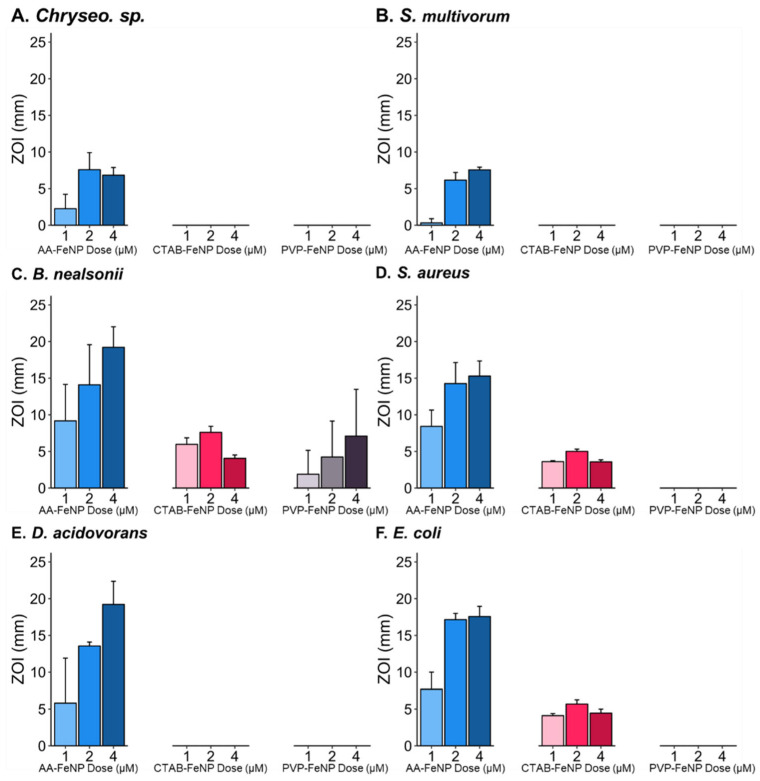
Analysis of nanoparticle efficacy and dose–response relationship for each species. Mean zone of inhibition (ZOI) values are compared across coated iron nanoparticle (FeNP) exposure dose for species (**A**) *Chryseobacterium* sp., (**B**) *S. multivorum*, (**C**) *B. nealsonii*, (**D**) *S. aureus*, (**E**) *D. acidovorans*, and (**F**) *E. coli*. Intra-species responses showed similarity between 2 and 4 µM doses. See Table 4 and Table 5 for statistical analyses of these data.

Increasing AA-FeNP dose revealed a diminishing effect across all taxa; little increase in efficacy was observed in the intra-species comparison between the 2 and 4 µM doses (Figure 4). Three bacteria exhibited an effect after exposure to CTAB-FeNP: *B. nealsonii*, *S. aureus*, and *E. coli*. *B. nealsonii* was the only species inhibited by PVP-FeNPs.

### 3.5. Species Susceptibility to Coated Iron Nanoparticles

**Figure 5:**
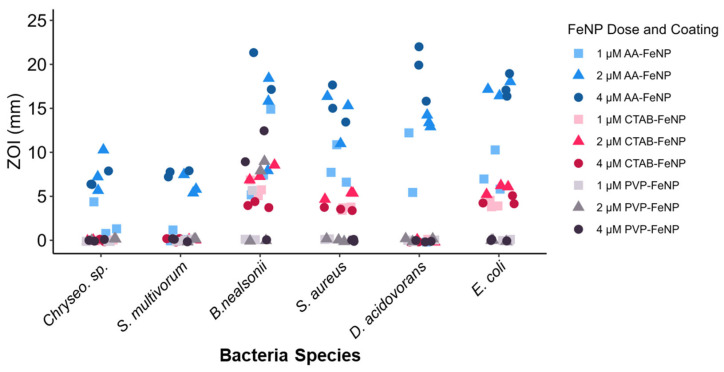
Analysis of the antimicrobial response of each nanoparticle treatment grouped by bacteria species. Zone of inhibition (ZOI) values show differences in response across species. Results show that *Chryseobacterium* sp. and *S. multivorum* are less susceptible than other species in the study. See Table 4 and Table 5 for statistical analyses of these data.

The degree of susceptibility to FeNP treatments varied by bacterial species (Figure 5). Species-specific analysis of efficacy data grouped across nanoparticle coating and concentration revealed that *B. nealsonii* and *E. coli* were most susceptible to collective treatments. The two taxa from the phylum Bacteroidota (*Chryseobacterium* sp. and *S. multivorum*) were the least susceptible. *S. aureus* and *D. acidovorans* showed intermediate responses.

Characteristic traits of each taxon may determine the species’ response to nanoparticle treatments. Bacillota and Pseudomonadota showed similar responses to FeNP treatments. The two most susceptible species of these phyla were *B. nealsonii* and *E. coli*. Previously, *B. nealsonii* was shown to be resistant to oxidative stress [87] but was affected by bare zero-valent iron nanoparticles more than other taxa, indicating that another mode of action other than oxidative stress may be at play [37]. *E. coli* is known to have metal-resistant genes and may be able to tolerate higher concentrations of intracellular ions from other metals [54]. However, previous studies have shown that bare zero-valent iron disrupts the *E. coli* membrane, producing ROS [41]. Gammaproteobacteria (the respective phylogenetic class of *E. coli*) abundance decreased in soil samples treated with bare zero-valent iron nanoparticles, supporting this study’s findings [37]. The mean order of susceptibility based on the disc assay is *B. nealsonii* > *E. coli* > *S. aureus* > *D. acidovorans* > *Chryseobacterium* sp. > *S. multivorum*.

**Figure 6:**
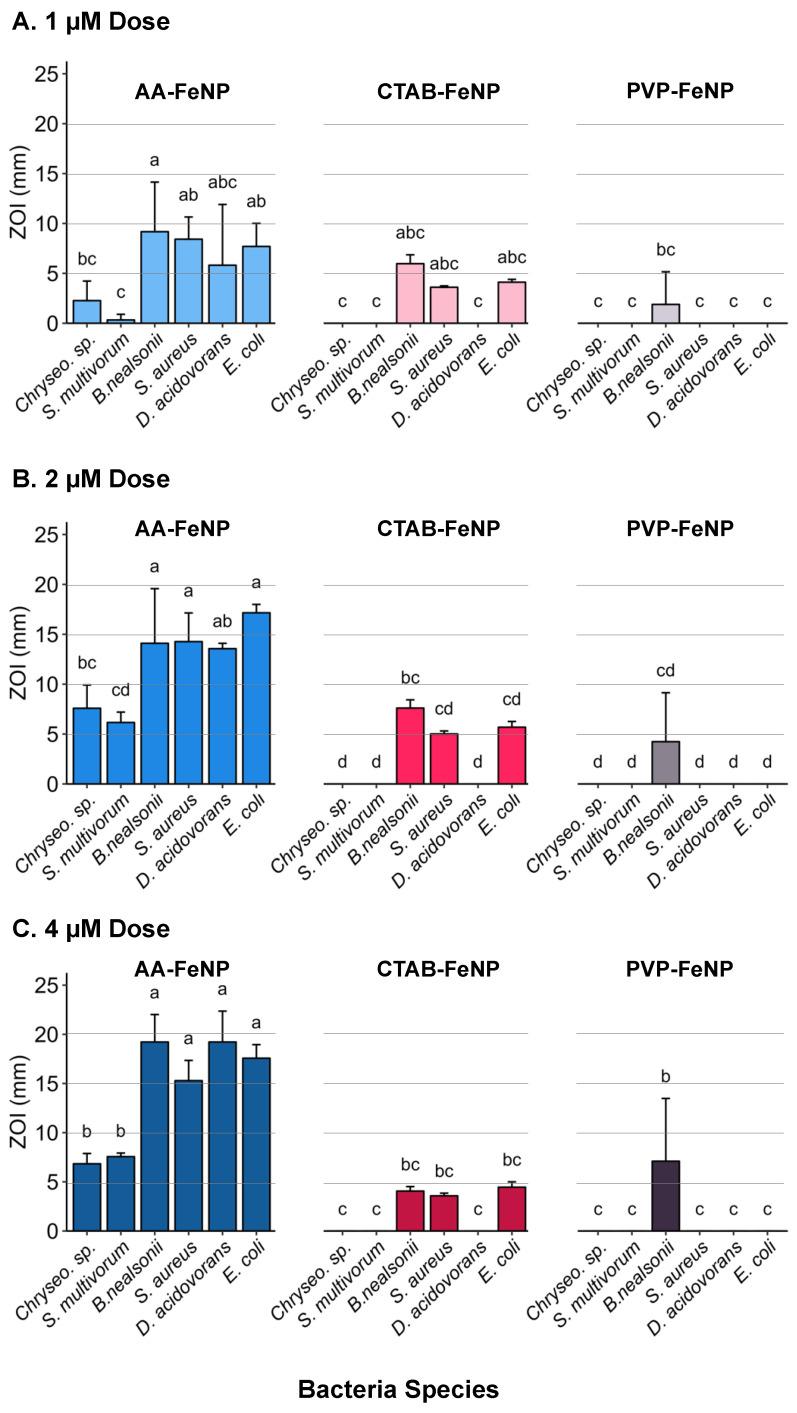
Analysis of bacteria species, surface coating, and dose. The comparison of mean zone of inhibition (ZOI) values among species after iron nanoparticle (FeNP) inoculation for (**A**) 1 µM exposure dose, (**B**) 2 µM exposure dose, and (**C**) 4 µM exposure dose. Results show *Chryseobacterium* sp. and *S. multivorum* had consistently lower responses across coatings than other species, while the response of *D. acidovorans* varied by coating and dose. See Table 4 and Table 5 for statistical analyses of these data. Data for different letters varied significantly.

Inter-species comparison across coatings revealed differential responses between species and surface coatings (Figure 6). The two species with the lowest response to the AA-FeNP treatment, *Chryseobacterium* sp. and *S. multivorum*, did not show responses to either CTAB-FeNPs or PVP-FeNPs, indicating that taxa-specific responses enabling relative resistance to one nanoparticle type are consistent across capping agents.

Bacteria most susceptible to AA-FeNPs were also susceptible to CTAB-FeNPs. An exception was *D. acidovorans*, which had a relatively large ZOI in response to the AA-FeNP treatment but did not show a response to either the CTAB-FeNP or PVP-FeNP treatment. Interestingly, *D. acidovorans* is known to have porin proteins that strongly select against cations due to positively charged amino acids surrounding the porin’s constriction site [88,89]. This supports the thought that ascorbic acid adds antimicrobial properties to the core. At the same time, *D. acidovorans* can select against cation entry; ascorbic acid is sufficient to cause a large ZOI in AA-FeNP treatments. The difference in effects between AA-FeNPs and CTAB-FeNPs in *D. acidovorans* may be due to the different modes of action between coatings. Studies are needed to elucidate the role of acid-based and surfactant-containing capping agents in conjunction with and separate from metallic cores. The coating-specific differences observed across taxa support the idea of a “designer nanoparticle” framework, indicating that nanoparticles could be designed to target taxa of interest.

An antibacterial potency threshold can also be proposed irrespective of dose for each FeNP type. In this scenario, the surface coating modifies the potency of the FeNP core, with less potent FeNP types affecting only the most sensitive species. This idea is seen most clearly in Panel A (1 µM dose), where the same three species with the highest responses to the AA-FeNP treatment likewise responded to the CTAB-FeNP treatment (i.e., *B. nealsonii*, *S. aureus*, and *E.coli*). The species with the highest response to CTAB-FeNP is also the only species with a response to PVP-FeNP (i.e., *B. nealsonii*). Here, the three most susceptible species with ZOI values above the proposed potency “threshold” for AA-FeNP were also above the “threshold” for the CTAB-FeNP, although less inhibition was observed. Nonetheless, *B. nealsonii*, the most sensitive species to all FeNP treatments, was the only bacteria to respond to PVP-FeNP, indicating that its sensitivity was above the proposed threshold for all coatings. Additional studies should be designed to investigate the thresholds related to the species, dose, and coating.

### 3.6. Statistical Data Analysis

The antibacterial nanotherapeutic field lacks a statistical framework for comparing efficacy across diverse treatments. Often, datasets generated to compare diverse treatments are non-normal, making it difficult to assess efficacy differences due to experimental factors without violating assumptions for standard analytical tests. The statistical framework used in this study enabled comparison across multiple variables and provided a rich analysis of the effects of FeNP coating, dose, and species.

The coefficient estimates for the GLMM model are shown in Table 4. Our analysis found a significant interaction between the dose and the AA coating, suggesting these factors are correlated in inhibiting bacterial growth. Since all the covariates are indicator variables for treatments, the coefficient estimates are interpreted as an estimated average multiplicative increase in inhibition relative to the base treatment. For example, since the estimated average inhibition for the base treatment (Coating = CTAB, Species = *S. multivorum*, and Dose = 1 µM) is a ZOI of 1.61 mm, the model suggests that the estimated average inhibition for Coating = AA, Species = *S. aureus*, and Dose = 2 mM would be 1.61 × 1.69 × 2.49 × 1.34 × 1.58 = 14.34 mm. The various expected inhibitions for different treatments can be explored using this framework.

Table 5 shows the results of the ordinal regression model. This analysis also found a significant interaction between the dose and the AA coating. One key difference between this model and the GLMM is that the coefficients represent the change in the odds ratio for each treatment. For example, on average, the AA-FeNPs are 160 times more likely to induce a greater inhibition than CTAB-FeNP, holding all other treatments fixed. Results from this model were consistent with GLMM output.

### 3.7. Phylogenetic Considerations for Iron Nanoparticle Efficacy

**Figure 7:**
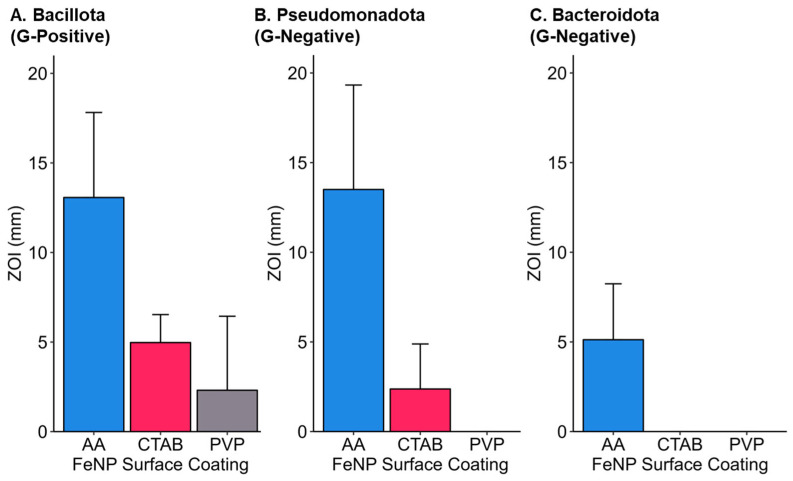
Inhibition grouped by phyla and nanoparticle coating. Each panel shows the zone of inhibition (ZOI) measurement value versus iron nanoparticle (FeNP) coating for two species from phylum (**A**) Bacillota, (**B**) Pseudomonadota, and (**C**) Bacteroidota. Phylum Bacillota is a Gram-positive bacterium, while Pseudomonadota and Bacteroidota are Gram-negative.

Phylogenetic characteristics may play a role in determining nanoparticle antibacterial effects. Grouping all inhibition data by phyla shows differences in means between bacteria phyla (Figure 7). Collective Bacteroidota ZOI was lower than both Bacillota and Pseudomonadota. Bacillota, the Gram-positive phyla in the study, had the largest mean ZOI. Grouping all data by cell wall structure revealed differences between Gram-positive and Gram-negative responses. Various studies have shown Gram-positive bacteria to be more susceptible to nanoparticle treatments than Gram-negative [90,91,92], although this is not always observed [11,93,94]. However, as the complete mechanism of antibacterial nanoparticle action is yet to be discovered, it is unclear how different nanoparticle cores and coatings affect different taxonomic groupings of bacteria, especially for zero-valent FeNPs, for which there has been little research. A previous study testing zero-valent iron clusters showed a preferential effect against Gram-positive bacteria over Gram-negative, similar to our findings of capped, zero-valent FeNP [95]. While some research has suggested that the porosity in the peptidoglycan cell wall allows for the translocation of nanoparticles, recent research indicates that the same size threshold that begins to cause antibacterial effects is the same for Gram-positive as Gram-negative bacteria, suggesting a different mechanism [96]. Another reason for the differential effects between Gram-positive and Gram-negative bacteria stems from the charge of the bacteria membrane. Although bacteria membranes are most commonly negatively charged, some species have different charge magnitudes due to membrane composition, which may be associated with taxonomic groups of bacteria [90,97,98]. Differences in bacteria membrane composition influence electrostatic and non-polar interactions, causing varied susceptibility of bacteria groups to nanoparticle treatments [8,82,99]. Multiple biochemical and physicochemical mechanisms may be at work to cause differential antibacterial effects across diverse bacteria.

### 3.8. Implications for Environmental and Biomedical Applications

In addition to their potential utilization as antimicrobial agents, nanoparticles with metal cores have been proposed for diverse environmental applications, including treatments for plant disease, targeted/slow-release fertilizers, and general-use pesticides [100,101,102]. The ability of FeNPs to adsorb toxic pollutants, such as heavy metals, has led to their application for remediation efforts [103,104,105,106,107]. However, the collateral effects of FeNPs on the environment are understudied, including their influence on microbial communities [108]. Metal nanoparticles may persist in the environment for long periods and could degrade soil quality by harming beneficial microbial communities and reducing crop growth and production [109,110]. Previous research has shown microbial community shifts in response to FeNP treatment, highlighting this concern [37,111]. It is unknown if trace metal ions leached from particle surfaces are used as micronutrients, thus increasing microbial enzyme function and growth, or if the exposure reduces collective metabolic function due to toxic effects.

Iron nanoparticles can effectively kill bacteria while also scavenging contaminants in soils. Therefore, the dosage of iron nanoparticles must be optimized to unlock their full potential in agricultural applications. In this study, our findings indicate that some bacteria found in the soil are affected by low concentrations of FeNP treatments, including taxa known to have plant-benefiting traits. Optimization of FeNP treatment concentration has the potential to balance practical remediation benefits against adverse collateral effects [112]. Our work also shows that the antibacterial effects of FeNPs can be moderated through a capping agent, as PVP-FeNP treatments yielded little antibacterial response. Future studies should explore the role of nanoparticle coatings in environmental applications to minimize collateral damage to non-target organisms. FeNPs may offer several remediation benefits, but further research is necessary before applying them in large-scale environments.

FeNPs have many potential applications in biomedical research, but clinical use is limited due to a lack of understanding of FeNP mechanisms [113]. Although ion leaching and resulting cellular toxicity were found during previous investigations of the in vitro use of FeNPs [114,115,116], the potential of using a capping agent to reduce toxicity while retaining ferromagnetic capability should be investigated [117,118,119]. Results from this study show many potential biomedical applications for capped zero-valent FeNPs. Our work demonstrates that FeNPs capped with an antioxidant, such as L-ascorbic acid, induce significant antibacterial effects, which should be investigated further for applications in vivo. PVP-FeNPs were shown to have minimal antibacterial action. This suggests the capping agent may prevent ion leaching or harmful interaction with bacterial membranes. Based on these results, this capping agent may also prevent harmful effects when applied in vivo in clinical settings. Capping agents may be tailorable to maintain chemical properties of interest (ferromagnetism or antibacterial properties) while preventing cellular damage to human cells.

Our work shows that capped FeNPs have antibacterial effects against bacterial taxa known for developing antibacterial resistance. Capped nanoparticles show promise as a solution to antibacterial resistance, as their multi-mechanistic mode of action and ability to be tailored allows them to bypass many mechanisms of bacterial resistance [15]. The differential effects of capped FeNP against diverse species indicate that nanoparticles could be developed to target specific bacterial taxa of interest. However, published studies lack tests on diverse bacteria, creating difficulty in understanding species-specific mechanisms. This research clarifies avenues for further research, highlighting the benefits of capped FeNPs and their effects on diverse bacterial species.

## 4. Conclusions

Our research shows that bacteria FeNPs have significant antibacterial effects against diverse bacterial taxa. Through the statistical framework developed in this study, we determined that the capping agent significantly influences nanoparticle antibacterial efficacy. Significant interactions between dose and coating differentially affect bacteria species, indicating that taxa-specific characteristics may govern the antibacterial effectiveness of nanoparticle core–coating combinations. This supports the “designer nanoparticle” ideology, suggesting that core and capping agents can be modified to increase targeting ability. More research is needed to elucidate mechanisms influencing bacterial susceptibility to better design nano-antibiotics that are safe for the environment and effective against bacteria strains of interest. Implementing robust statistical models is necessary to understand complex treatments’ effects, interactions, and collective influence on antibacterial response. The antibacterial effects of FeNPs on agriculturally relevant taxa should be considered before implementing environmental applications. This research strongly supports the feasibility of designing nano-antibiotics for targeting bacterial populations of interest, which will significantly increase the efficacy of FeNP applications in environmental and biomedical fields.

## Figures and Tables

**Table 1:** The six bacterial taxa used in this study. This table includes information on cell wall type and morphology, the environmental compartment for which it is commonly found, and references related to biological structure and function.

Bacteria	Phylum	Class	Cell Wall and Morphology	Environmental Compartment	References
*Bacillus nealsonii*	Bacillota	Bacilli	Gram-positive rods	Sediment	[47]
*Chryseobacterium* sp.	Bacteroidota	Flavobacteriia	Gram-negative rods	Human tissue, Soil, Water, Plants	[48,49,50]
*Delftia acidovorans*	Pseudomonadota	Betaproteobacteria	Gram-negative rods	Human tissue, Soil, Water, Plants	[51,52,53]
*Escherichia coli*	Pseudomonadota	Gammaproteobacteria	Gram-negative rods	Human tissue, Soil, Water, Plants	[54,55,56]
*Sphingobacterium multivorum*	Bacteroidota	Sphingobacteriia	Gram-negative rods	Human tissue, Soil, Water	[57,58,59,60]
*Staphylococcus aureus*	Bacillota	Bacilli	Gram-positive coccus	Human tissue, Livestock	[61,62]

**Table 2:** Variables and descriptions used in the GLMM analysis of antibacterial action.

Symbol	Variable Explanation
α	Intercept term; Estimated logged mean inhibition for Coating = CTAB on Species = *S. multivorum* using Dose = 1 µM
$\beta_{i}$	Coefficient for each covariate; Estimated multiplicative factor for log mean inhibition
$x_{1}$	Indicator variable for Coating = AA, $x_{1}\in \left(0,1\right)$
$x_{2}$	Indicator variable for Species = *Chryseobacterium* sp., $x_{2}\in \left(0,1\right)$
$x_{3}$	Indicator variable for Species = *S. aureus*, $x_{3}\in \left(0,1\right)$
$x_{4}$	Indicator variable for Species = *D. acidovorans*, $x_{4}\in \left(0,1\right)$
$x_{5}$	Indicator variable for Species = *E. coli*, $x_{5}\in \left(0,1\right)$
$x_{6}$	Indicator variable for Species = *B. nealsonii*, $x_{6}\in \left(0,1\right)$
$x_{7}$	Indicator variable for Dose = 2 µM, $x_{7}\in \left(0,1\right)$
$x_{8}$	Indicator variable for Dose = 4 µM, $x_{8}\in \left(0,1\right)$

**Table 3:** Results of logistical regression showing the relative probability of a treatment inducing an antibacterial effect compared to the PVP-FeNP treatment. Odds ratios reveal that CTAB-FeNP and AA-FeNP are estimated to be 10 times and 167 times more likely to have a non-zero inhibition than PVP-FeNP, respectively. The *p*-value indicates statistical significance. NA stands for not applicable.

Coating	Odds Ratio	*p*-Value
PVP-FeNP	1	NA
CTAB-FeNP	10.0	<0.001
AA-FeNP	167	<0.001

**Table 4:** Exponentiated coefficients for GLMM using *S. multivorum* exposed to CTAB-FeNP at a dose of 1 µM as the base treatment. *p*-values less than 0.05 are considered significant. NA stands for not applicable.

Model	Coefficient	Estimate	*p*-Value
Gamma	Intercept	1.6079	0.0015
	Coating = AA	1.6944	<0.001
	Species = *Chryseo.* sp.	1.0607	0.668
	Species = *S. aureus*	2.4879	<0.001
	Species = *D. acidovorans*	2.7729	<0.001
	Species = *E. coli*	2.7901	<0.001
	Species = *B. nealsonii*	3.1417	<0.001
	Dose = 2	1.3422	0.0208
	Dose = 4	0.8969	0.3933
	Coating = AA × Dose = 2	1.5756	0.0044
	Coating = AA × Dose = 4	2.7055	<0.001
ZI	Intercept	0.3896	<0.001
Dispersion	Intercept	13.7187	NA

**Table 5:** Ordinal model output for statistical comparison using *S. multivorum* exposed to CTAB-FeNP at a dose of 1 µM as the base treatment. *p*-values represent significance in the probability that the treatment differs from the baseline. The estimate gives the degree of increase and decrease in antibacterial response, with estimate values less than one indicating a decreased response and values greater than one indicating an increased response. The *p*-values <0.05 are considered significant.

Variable	Estimate (Exponentiated)	*p*-Value
Coating = AA	160.4317	<0.0001
Species = *Chryseo.* sp.	1.8263	<0.0001
Species = *S. aureus*	883.8004	<0.0001
Species = *D. acidovorans*	81.9498	0.1204
Species = *E. coli*	3051.8002	0.0007
Species = *B. nealsonii*	7912.0398	<0.0001
Dose = 2	7.6041	0.0064
Dose = 4	0.5471	0.3816
Coating = AA × Dose = 2	43.2076	0.0002
Coating = AA × Dose = 4	2628.5285	<0.0001

## Data Availability

The original contributions presented in the study are included in the article/Appendix A, further inquiries can be directed to the corresponding author.

## Appendix A.

### Appendix A.1. Iron Nanoparticle Transmission Electron Microscopy

Transmission electron microscopy (TEM) was performed for morphological characterization (JEOL, JEM-1010, TEM, Tokyo, Japan). A drop of the prepared nanoparticle suspension was added to the surface of carbon-coated copper grids (Electron Microscopy Sciences; Hartfield, PA, USA) and dried in a hot air oven at 160 °C. The dried TEM grids were then viewed on the TEM at an accelerating voltage of between 10 and 100 kV.

### Appendix A.2. Response Variable Distribution

Analysis of response variable distributions by FeNP type revealed that the distributions of each nanoparticle coating are highly zero-inflated (Figure A1) and highly skewed right. No transformations were found to be stabilizing. Therefore, more than traditional statistical methods are required to conduct robust analysis, and alternative models are needed.

### Appendix A.3. Ordinal Model Assumptions

One way to validate ordinal model assumptions is to check if the odds ratio for each treatment level is proportional to one another (Figure A2). Within this experiment, the number of observations within each treatment group was too small to validate this assumption fully; however, proportional odds ratios were validated across factor levels. It has been determined that ordinal regression models are still very robust to this assumption, which makes it one of the safest models for drawing inferences on the zone of inhibition given different treatments [120]. All the empirical cumulative density functions (eCDF) under a logistic link for each group look reasonably proportional (roughly parallel), validating our assumption.
